# Every-Day Science

**Published:** 1888-01-07

**Authors:** 


### The Doctor's Pulpit.

### EVERY-DAY SCIENCE.

Many excellent people have a great dread of science and all its works and professors. They think it is going to uproot everything that is venerable in religion and morals, and to bring in an age of pure materialism. There is no necessary tendency in scientific discovery to undermine morals or to destroy religion. It is true that human traditions have often had to give place to newly-discovered facts, and that many devout believers in those traditions have been called upon to endure severe mental shocks on that account. But later generations have wondered, not only that people should have seen such danger to religion in the removal of traditions which never had any real relation to religion, but also how, among reasonable people, such traditions could ever have come to be accepted and believed at all.

A great many persons of fair intelligence never take the trouble to understand what science is. They have a vague idea that it is a condition of mind which delights in the invention of all kinds of new and dangerous explosives—explosives which can be used with deadly effect, not only in the material but also in the moral world. A scientific man these good people regard as a sort of diminutive Lucifer, who finds a fierce pleasure in defying everybody all round and in asserting things which make the hair of conventional orthodoxy stand on end. Some scientific men no doubt have been of this kind, and so the whole class has suffered. But in science itself there is not and cannot be anything contradictory to sound morals or to reasonable religion. People should remember that science does not make facts—it merely discovers and affirms them. All the discoveries of recent years have resulted in the bringing to light of facts which have been waiting to be brought to light since the days of Aristotle and thousands of years before that time. The facts are not only there now, but they always have been there: the mere discovery of them makes no changes in the constitution and order of the world or in the moral and spiritual nature of man.

Timid men and women of religious instincts need not fear science at all. They should seek an introduction to her, shake hands with her, and learn to regard her as a friend. She really is a friend, though sometimes at the first blush she has so strange an appearance that, like children or cattle, we are all apt to be a little scared. But if it be borne in mind, as we have already said, that scientific workers can never alter by one iota the "constitution and course of nature," as Butler has it, but can only make us see every department of nature more clearly, and understand better what is going on in her laboratories, the fear of science and her army should entirely disappear.

Science, be it remembered, is not a plaything, or a mere part of an educational course as Greek and geometry are to many men. It is something of which highly practical uses can be made. He who has but a little of it has at any rate a farthing rushlight wherewith to illuminate the darkness of a world full of mysteries; while he who has much may be said to live in the blaze of the noonday sun, as compared with him who has none. A very familiar illustration which occurs to us is the subject of domestic sanitation. At the present moment a medical man of our acquaintance is attending two children in one house who have membranous tonsillitis—a kind of diphtheria, in fact. A few weeks ago a young lady visitor in the same house was suffering in the same way. A few months before that the mother was laid up with the affection. Indeed, for seven years at least the house has seldom been free from illness for more than a few months, and the diseases have been generally either of the character named or typhoid fever. There is no doubt that the sanitary conditions of the house are bad. Every time the doctor has been called in he has asked about the drainage, &c.; every time it has been promised that a thorough overhauling should be given to it; every time, there is the best reason to believe, the promise has been forgotten; and the results have been such as stated. That no deaths have occurred is an occasion for considerable surprise.

Now a very little really intelligent understanding of scientific facts might have prevented almost every one of the outbreaks of illness in this particular house. In that case how much labour, how much anxiety, how much money might have been saved. The doctor has spared no trouble or earnestness in insisting upon what he believes to have been the preventable causes of the outbreaks; but if people will not learn and will not believe, there is a temptation to let them go their own way.

The present phase of medical opinion on the "microbe" question may seem to belong exclusively to the medical profession, and in one sense undoubtedly it does. But the general public have an interest in such a subject which is vital. They cannot help to elucidate the question either of the cause or cure of phthisis; but they may do a very great deal towards its prevention in their own families. If it be true, as seems probable, that a peculiar microbe is the cause of consumption, and that this microbe is harmless under certain conditions and dangerous under others, what a light is here thrown on circumstances which have often seemed altogether too mysterious for explanation or modification! A consumptive patient will now be rightly regarded as a dangerous source of infection within certain limits. Not probably dangerous to those with sound and healthy lungs, unless they remain unduly long in the sick room, and neglect the ordinary means of maintaining vigorous health; but dangerous to those who have any hereditary or acquired tendency to phthisis, and who should therefore, in every case where circumstances will permit, avoid as much as possible all contact with persons suffering from the active form of the disease.

If it be also true, as modern observation and research go to show, that certain circumstances—such as insufficient or ill-chosen food, a cold, damp atmosphere, bad air, and all the conditions depressing to the general health, are peculiarly conducive to the development of phthisis in certain persons; whilst, on the other hand, high altitudes, where the air is clear and bracing, life lived in the open, a wholesome and nourishing diet, with other attendant advantages, are markedly antagonistic to the growth and increase of the microbe; if these things be so, how much may parents, even of moderate means, do to secure for their children every possible circumstance favourable to them in their struggle with so mortal an enemy. This is a kind of science perfectly easy to understand, and the lessons it teaches not only may but ought to be learnt by every one who has children or relatives in any way predisposed to phthisical affections. Vast numbers of lives might be saved annually if parents and guardians would try to appreciate the significance of the observations and conclusions of competent observers, which are here indicated; and, recognising how much easier it is to fight one regiment than a hundred, would, at the very commencement of unfavourable symptoms, place those whom they love in such circumstances as would tend to prevent any multiplication of the microbe regiments whatever. The discoveries of science do not belong to a class, but to the world. Those who make them have no pleasure in their being kept secret, but rather wish them to be proclaimed upon the housetops. The interests of both medical and other sciences and those of the public are not antagonistic, but identical. Every physician who has true sympathy with his fellows, and with the scientific progress of the time, cannot but rejoice at the general attention given on all hands to those great questions which affect the life and health of the community.

